# Visual Rehabilitation of Bilateral Keratoconus With Customized Scleral Lenses: A Case Report

**DOI:** 10.7759/cureus.113637

**Published:** 2026-07-30

**Authors:** Nabila Y Al-Tamimi, Mohammed Amer, Fadila Nour Elrihen Graini, Ramdas Gore

**Affiliations:** 1 Ophthalmology Department, Hamad Medical Corporation, Doha, QAT; 2 College of Medicine, Qatar University, Doha, QAT

**Keywords:** corneal ectasia, corneal tomography, keratoconus, pentacam, scleral lenses, visual rehabilitation

## Abstract

The clinical management and visual rehabilitation of a 24-year-old female with bilateral keratoconus (OD > OS) successfully fitted with scleral lenses is presented. A comprehensive ophthalmic evaluation was performed, including keratometry, corneal topography using Pentacam imaging, and objective and subjective refraction. Objective refraction revealed +3.00 −5.00 × 105 in OD and +1.50 −3.00 × 080 in OS. The final spectacle prescription was +2.00 −4.50 × 105 in OD, achieving a visual acuity of 6/9− (20/30−), and +0.75 −3.00 × 080 in OS, achieving 6/6− (20/20−). Diagnostic scleral lenses were selected based on sagittal depth, guided by Kmax values. Baseline unaided visual acuity was 6/24 (20/80) in the right eye (OD) and 6/18 (20/60) in the left eye (OS), with near vision of N6 in both eyes (OU), since the patient had no current spectacles. With scleral lenses, best-corrected visual acuity improved to 6/6 (20/20) in both eyes (OU). Topographic analysis revealed advanced ectatic changes in OD (Kmax: 70.80 D; enhanced ectasia score: 17.99 D). The lenses demonstrated appropriate vault, limbal clearance, and uniform scleral alignment, ensuring stable centration, comfort, and protection of the ocular surface. Scleral lenses provided effective visual rehabilitation in advanced keratoconus, restoring 6/6 (20/20) vision bilaterally while preserving corneal health. This case highlights the clinical value of scleral lenses as a reliable approach for managing irregular corneas and progressive ectasia.

## Introduction

Keratoconus is a progressive, non-inflammatory corneal ectasia characterized by corneal thinning and protrusion, leading to irregular astigmatism and significant visual impairment. Although historically considered a rare condition, its prevalence is notably higher in the Middle East compared to global averages. Recent studies report prevalence rates of up to 4.79% in Saudi Arabia, with similarly elevated figures in neighboring Gulf countries, including Qatar, attributed to genetic predisposition, environmental factors, and high rates of consanguinity [1,2]. In Qatar, although specific large-scale epidemiological studies remain limited, anecdotal and clinical observations suggest a rising trend, particularly among young adults seeking refractive surgery consultations [3,4]. Early detection is vital, as modern diagnostic tools, such as corneal topography and Purkinje image assessment, allow for timely interventions. Management strategies, including scleral contact lenses and corneal cross-linking (CXL), not only improve visual function but also halt disease progression, reducing the need for more invasive procedures such as corneal transplantation [5].

This case report highlights the clinical assessment and scleral lens fitting in a patient with keratoconus, emphasizing the importance of tailored management in the Middle Eastern context.

## Case presentation

Initial visit: day 1

A 24-year-old Qatari female presented to the Optometry Contact Lens Clinic with complaints of decreased and blurred vision in both eyes, reporting difficulty achieving satisfactory vision. She expressed interest in resuming contact lens wear, having previously worn rigid gas permeable lenses. The patient was diagnosed with bilateral keratoconus in 2018 at Al Wakrah Health Center and subsequently underwent corneal collagen CXL with riboflavin in both eyes at Hamad Medical Corporation later that year.

The patient has no systemic health issues or undermedication. Her medical history was unremarkable; however, she reported a positive family history of keratoconus in her younger brother, and she expressed interest in resuming wearing contact lenses, as she came for contact lens evaluation and fitting.

Upon examination, unaided visual acuity was 6/24 (20/80) in the right eye (OD), 6/18 (20/60) in the left eye (OS), and 6/18 (20/60) binocularly (OU) with near vision of N6 in both eyes (OU) as the patient had no current spectacles. Best spectacle-corrected visual acuity improved to 6/18+ (20/60+) in the right eye (OD), 6/18 (20/60) in the left eye (OS), and 6/18+(20/60+) in both eyes (OU). Objective refraction revealed +3.00 −5.00 × 105 in OD and +1.50 −3.00 × 080 in OS. The final spectacle prescription was +2.00 −4.50 × 105 for OD VA 6/9−(20/30−) and +0.75 −3.00 × 080 for OS VA 6/6−( 20/20-).

Slit-lamp examination

On slit-lamp biomicroscopy, the ocular adnexa, including the lids, lashes, conjunctiva, and iris, appeared normal. Using an optic section, signs of keratoconus were observed in both eyes, characterized by localized inferotemporal thinning and an irregular corneal surface. Meibomian gland dysfunction was graded as G1 bilaterally, accompanied by mild dry eye changes and papillae grade 1 in the upper and lower eyelids for both eyes (OU). Following instillation of 1 mg fluorescein sodium moistened with normal saline, corneal staining was detected as G1. The tear break-up time measured 14 seconds in the right eye (OD) and 14 seconds in the left eye (OS).

Corneal tomography (Pentacam)

**Figure 1:**
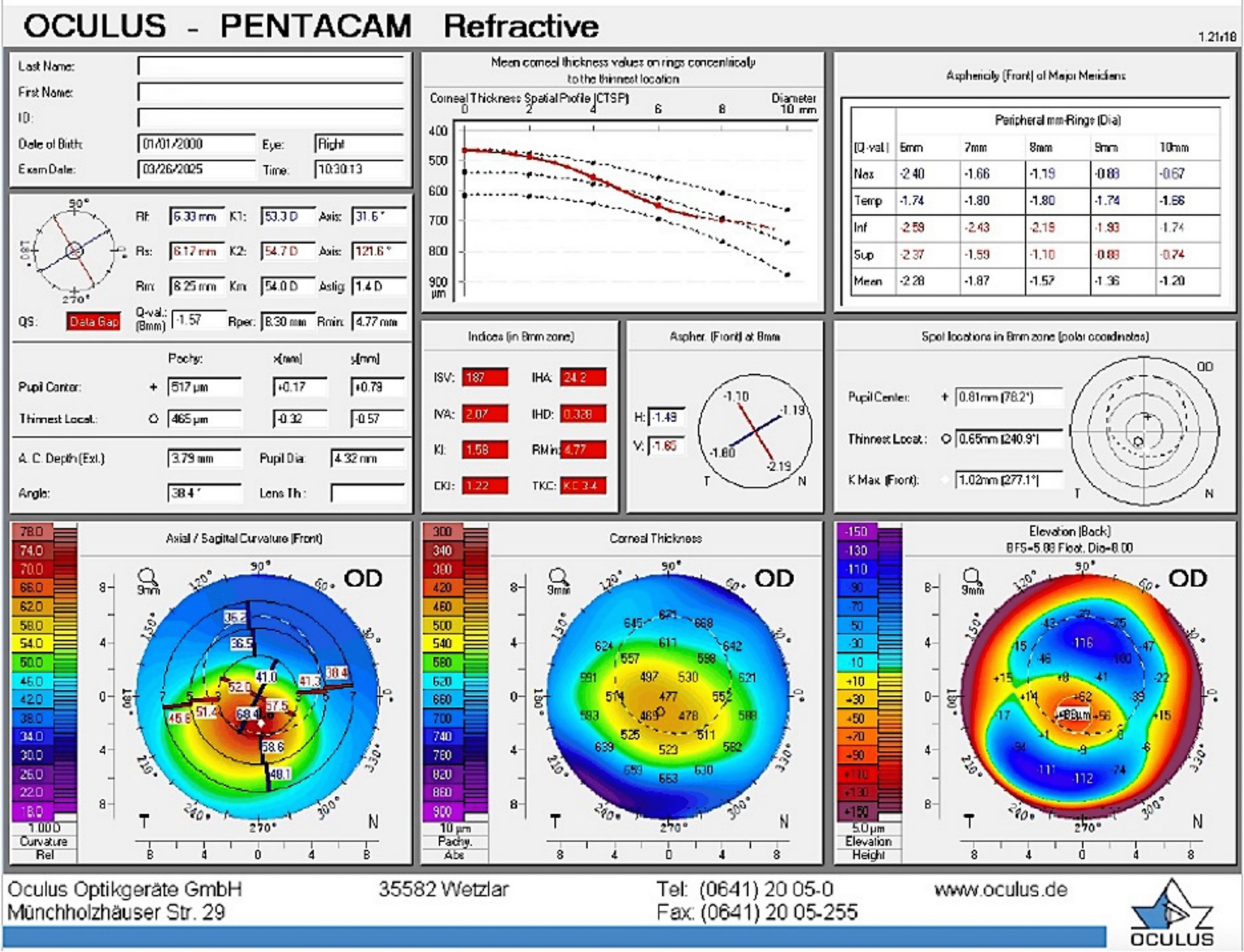
Oculus Pentacam imaging of the right eye (OD) in a 24-year-old female with keratoconus The cornea shows a base curve of 6.25 mm, mean power of 54.0 D, and astigmatism of 1.40 D, indicating marked steepening and irregularity. Diagnostic maps (corneal thickness spatial profile (CTSP), axial sagittal curvature, corneal thickness, and posterior elevation) reveal classic keratoconic features, including a maximum posterior elevation of +98 µm, confirming advanced ectasia essential for diagnosis, scleral lens fitting, and disease management.

**Figure 2:**
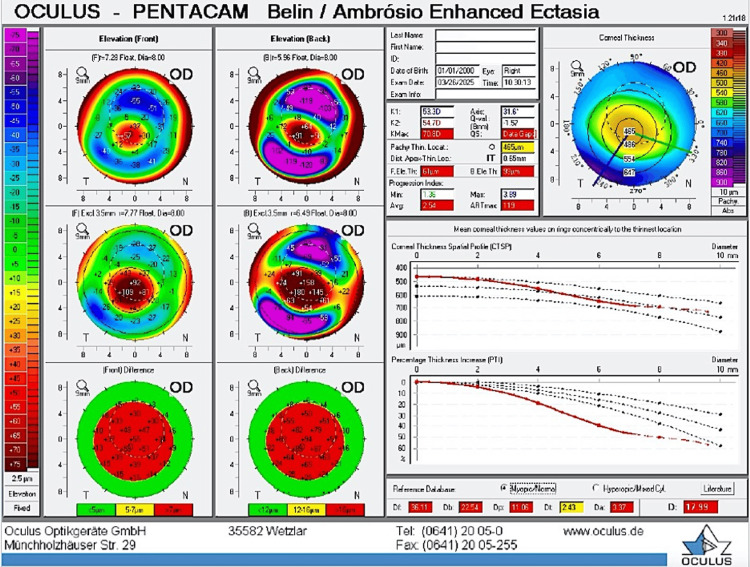
Belin/Ambrósio enhanced ectasia display (Oculus Pentacam) of the right eye (OD) Shows an elevated enhanced ectasia score of 17.99 d (normal < 1.6 d), confirming advanced keratoconus. The kmax value of 70.80 d and average progression index (API) of 2.54 (normal 0.8–1.1 mm) indicate marked corneal steepening and progressive thinning. Diagnostic maps (anterior/posterior elevation, corneal thickness, and corneal thickness spatial profile (CTSP)) reveal focal thinning with an S-shaped CTSP curve, consistent with significant biomechanical instability. Findings support advanced ectasia and guide management decisions, such as corneal cross-linking or scleral lens fitting.

**Figure 3:**
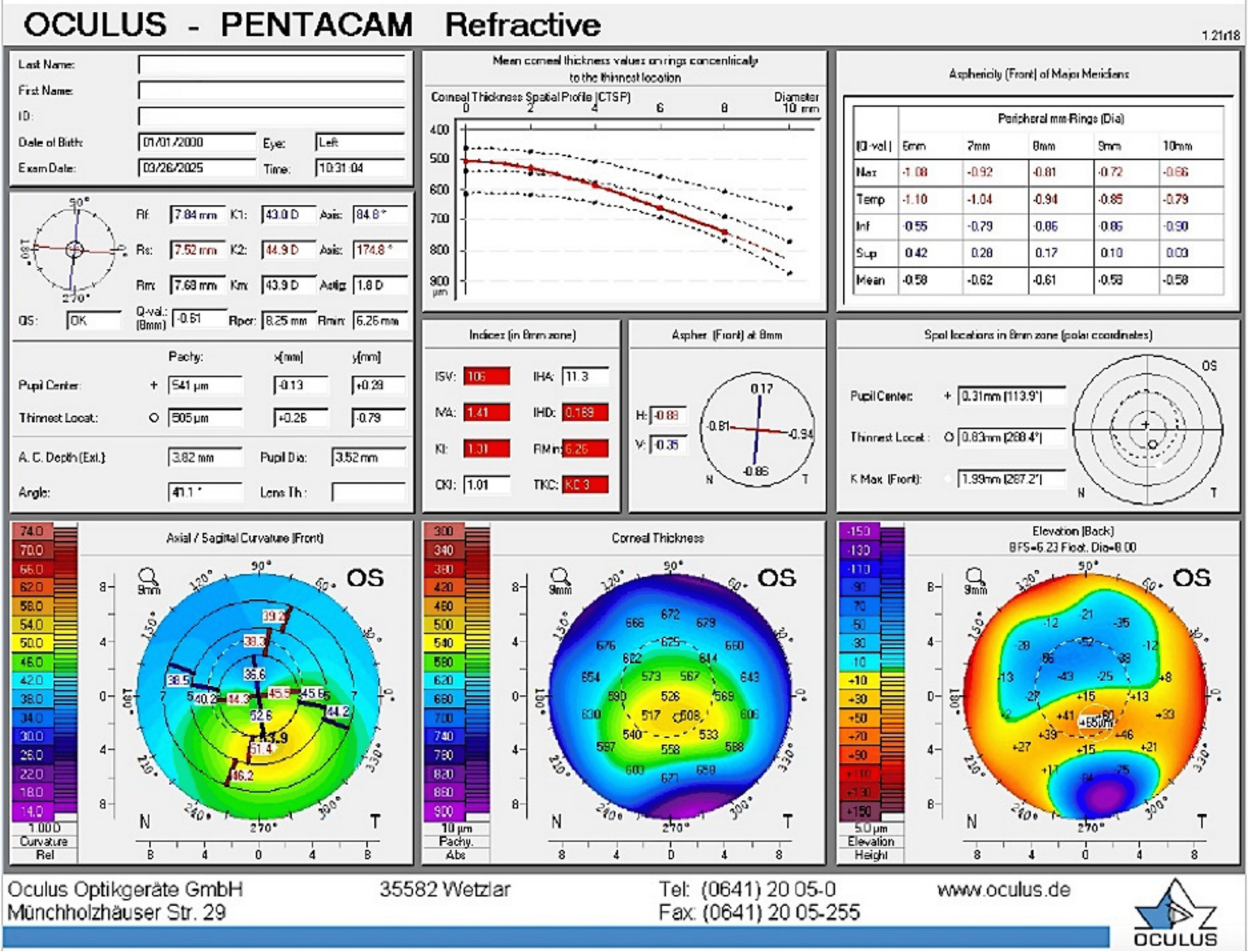
Oculus Pentacam imaging of the left eye (OS) in a 24-year-old female with keratoconus. Shows a base curve of 7.68 mm, mean corneal power of 43.9 D, and astigmatism of 1.8 D, consistent with mild-to-moderate ectatic changes. Posterior elevation of +65 µm and corneal thickness spatial profile (CTSP) findings indicate early keratoconus. Diagnostic maps (CTSP, axial sagittal curvature, corneal thickness, and posterior elevation) confirm early structural alterations, supporting precise diagnosis, monitoring, and personalized management.

**Figure 4:**
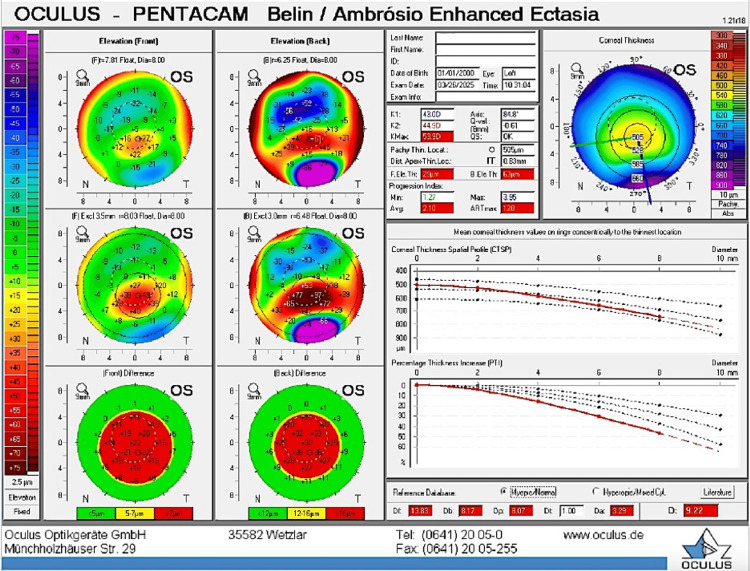
Belin/Ambrósio enhanced ectasia display (Oculus Pentacam) of the left eye (OS) Shows an enhanced ectasia score of 9.22 D and Kmax of 53.9 d, indicating significant ectatic changes. The average progression index (API) of 2.10 (normal 0.8–1.1) reflects progressive corneal thinning from center to periphery. Diagnostic maps, including anterior/posterior elevation, corneal thickness, and corneal thickness spatial profile (CTSP), demonstrate a quick slope curve consistent with advanced ectasia. Findings confirm keratoconus and highlight the role of tomographic analysis in early detection and individualized management.

Following up with keratometry measurements showed significantly steeper corneal curvature in the right eye (OD (average K: 56.25 D (6.00 mm), flat K: 53.75 D (6.29), Kmax: 70.80 D) compared to the left eye (OS average K: 46.50 D (7.24 mm), flat K: 46.00 D (7.35), Kmax: 53.90 D). In addition, we ordered a corneal topography to further investigate a cornea shape and to determine the proper contact lens fitting for this patient. We used OCULUS Pentacam, which demonstrated marked ectatic changes. In the right eye (OD), the base curve was 6.25 mm, with an average corneal power of 54.0 D and corneal astigmatism of 1.4 D. The posterior corneal elevation was notably elevated at +98 µm, classified as risky. The enhanced ectasia score was 17.99 D, and the average progression index (API) was 2.54, both highly abnormal. Similarly, the left eye (OS) showed a base curve of 7.68 mm, average corneal power of 43.9 D, and corneal astigmatism of 1.8 D, with posterior elevation of +65 µm, which also exceeds the threshold for risk. The enhanced ectasia score and API were 9.22 D and 2.10, respectively, indicating abnormal progression according to the Belin/Ambrósio Enhanced Ectasia Score established guidelines [6]. The figures below further explain the results of the patient during the visit (Figures 1-4). 

Contact lens fitting and specifications

The selection of a trial contact lens is guided primarily by the Kmax value and the morphology of the corneal cone according to the Optosoft fitting approach. For patients with a Kmax below 55.00 D, the fitting process typically begins with a trial lens having a 4300-micron sagittal depth (K4). In cases where the Kmax exceeds 55.00 D, a trial lens with a 4600-micron sagittal depth (K7) is preferred. In addition, the shape and location of the corneal cone are carefully considered to optimize lens fit, comfort, and visual performance.

For diagnostic contact lens fitting, the trial lens selection was guided by the Kmax values. As Kmax was above 55.00 D in the right eye (OD), a high sagittal depth lens (5100 µm) was selected, whereas a lower sagittal depth (4400 µm) lens was chosen for the left eye (OS). The lenses trialed were as follows during the patient visit.

Trial lenses and visual acuity (VA)

A scleral lens assessment was performed for both eyes. In the right eye (OD), an OPTOSOFT IS16 K10 scleral lens with a sagittal depth of 5100 µm, a base curve of 7.48 mm, a diameter of 16.00 mm, and a power of +0.25 D was fitted. The patient achieved a best-corrected visual acuity of 6/6− (20/20-) with this lens.

In the left eye (OS), an OPTOSOFT IS16 K5 scleral lens with a sagittal depth of 4400 µm, a base curve of 8.05 mm, a diameter of 16.00 mm, and a Plano power was fitted. With this lens, the patient also achieved a best-corrected visual acuity of 6/6− (20/20-). Overall, the selected scleral lenses provided excellent visual outcomes in both eyes.

Scleral contact lens trial: patient education, insertion technique, and fit evaluation

After determining the appropriate trial lens for the patient, the pros and cons of wearing scleral contact lenses were discussed. The lens insertion and fit evaluation then proceeded.

The lens was placed with the concave side facing upward on a sterile surface and filled with non-preserved saline solution, to which a small amount of fluorescein dye was added. The patient was instructed to tilt their head downward so that it was parallel to the floor. Using either a suction holder or the three-finger method (thumb, index, and middle fingers), the lens was gently applied to the central cornea, taking care to avoid air bubbles. Minor peripheral bubbles are generally inconsequential; however, central bubbles must be avoided as they can significantly impair vision and compromise the accuracy of the fit evaluation. If a central bubble is detected, the lens should be removed and reinserted. During this visit, the patient was compliant, and the lens was fitted successfully.

Following insertion, the lens was allowed to settle on the eye for approximately 20 minutes to achieve a stable position. The fitting assessment focused on three key parameters: apical clearance; optimal apical clearance should range between 250 and 350 µm, ensuring the lens vaults over the corneal apex without bearing directly on it. If the clearance is inadequate or excessive, lens selection can be adjusted by modifying sagittal depth: a deeper vault increases clearance, while a shallower vault decreases it.

**Figure 5:**
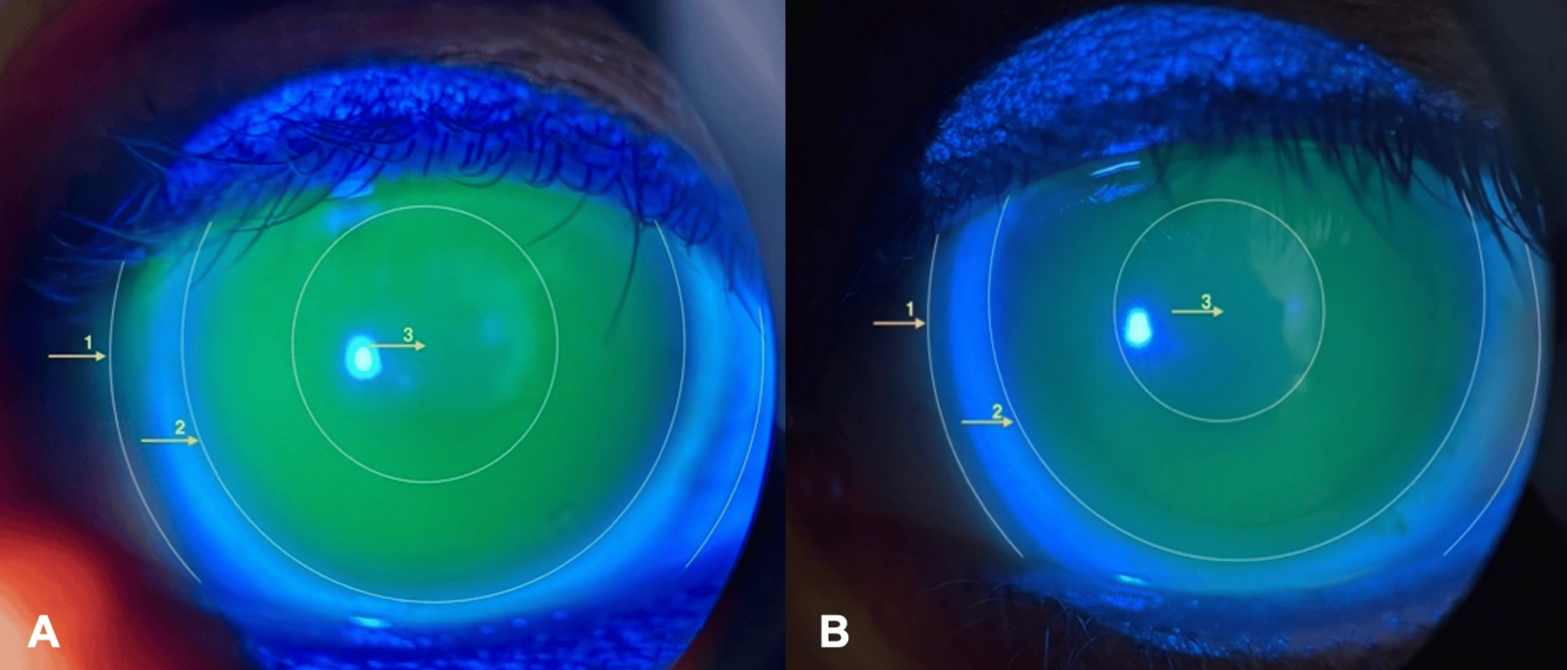
(a-b) Slit-lamp examination demonstrating scleral lens fitting in both eyes (OU) Illustration of the right eye (OD) on slit-lamp examination showing (1) alignment of the scleral landing zone, (2) adequate limbal clearance, and (3) good apical clearance, consistent with an optimal scleral lens fit. Illustration of the left eye (OS) on slit-lamp examination showing (1) alignment of the scleral landing zone, (2) adequate limbal clearance with adjustments of +1 step at 0° and 90°, and (3) good apical clearance, consistent with an optimal scleral lens fit.

**Figure 6:**
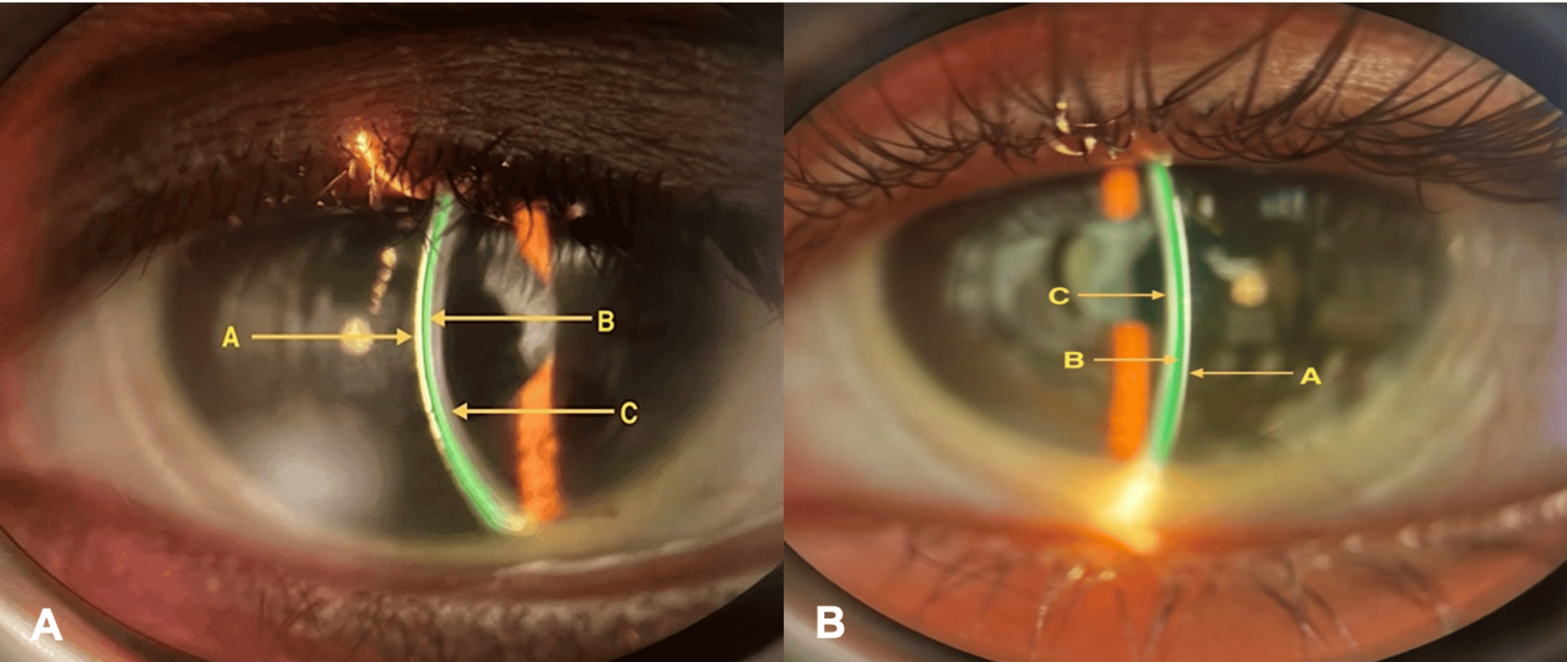
(a-b) Slit-lamp examination demonstrating optimal central vault and scleral lens clearance in both eyes (OU) Illustration of the right eye (OD). The central fit, evaluated using slit-lamp examination, demonstrates a gap between the lens and the cornea measuring approximately two-thirds of the thinnest corneal thickness (465 µm), indicating an optimal central vault. The arrows indicate: (A) scleral lens surface, (B) tear reservoir (tear vault), and (C) corneal surface confirming appropriate lens clearance and alignment. Illustration of the left eye (OS). On slit-lamp examination, the central fit shows a gap between the lens and the cornea measuring approximately two-thirds of the thinnest corneal thickness (505 µm), consistent with an optimal central vault. The arrows indicate (A) scleral lens surface, (B) tear reservoir (tear vault), and (C) corneal surface, confirming appropriate lens clearance and alignment.

Once settled, a slit-lamp biomicroscopy examination was performed to assess the fit of the scleral lens, showing lens position and alignment. In addition, the fluorescein pattern was assessed under cobalt blue light to evaluate apical clearance and confirm there was no corneal touch (Figures 5-6).

Scleral lens fit evaluation following a 20-minute settling

After a 20-minute settling period, the diagnostic scleral lenses were assessed for fit. The apical clearance measured 250-350 µm, and the limbal clearance was 50-150 µm. The scleral landing zone demonstrated even alignment at the cardinal positions (0°, 90°, 180°, and 270°). Lens selection and fitting strategy were customized according to the severity of the patient’s corneal ectasia, with the aims of optimizing visual rehabilitation and maintaining corneal health. The patient tolerated the diagnostic lenses well and achieved satisfactory visual outcomes, indicating a favorable prognosis for long-term scleral lens wear.

Fit evaluation parameters

Following insertion, the lens is allowed to settle on the eye for approximately 20 minutes to achieve a stable position. The fitting assessment focuses on three key parameters: apical clearance; the optimal apical clearance should range between 250 and 350 µm. This ensures that the lens vaults over the corneal apex without bearing directly on it. If the clearance is inadequate or excessive, lens selection can be adjusted by modifying sagittal depth. For example, increasing sagittal vault raises clearance, while decreasing it reduces clearance.

Apical clearance

The optimal apical clearance should range between 250 and 350 µm to ensure that the lens vaults over the corneal apex without bearing directly on it. If clearance is inadequate or excessive, sagittal depth can be modified accordingly. Increasing sagittal vault raises clearance, while decreasing it reduces clearance.

Limbal clearance

Limbal clearance should ideally range between 50 and 150 µm to protect the limbal stem cells while maintaining proper lens fit. If apical clearance is adequate but limbal clearance is insufficient, adjustments can be made in 25 µm increments. A “+” value denotes increased clearance, while a “-” value indicates reduced clearance. Custom adjustments are typically measured at a chord diameter of 11.80 mm.

Scleral landing zone (SLZ)

The peripheral landing zone of the lens should align evenly with the scleral surface in all four quadrants (0°, 90°, 180°, and 270°). Proper alignment ensures uniform pressure distribution and minimizes conjunctival compression or blanching. Adjustments are made in 25 µm increments: a “+” value flattens, while a “-” value steepens the periphery. Correct SLZ alignment promotes lens centration, stability, and long-term comfort.

Rotational orientation (RO) and axis compensation

Rotational orientation (RO) was assessed following lens stabilization, as accurate toric lens alignment is essential for optimal visual performance. The position of the reference mark on each lens was used to determine rotation. Clockwise rotation was recorded as a negative (−) value, whereas counterclockwise rotation was recorded as a positive (+) value.

In this case, the right eye (OD) demonstrated a rotational orientation of 105° (clockwise, recorded as −), and the left eye (OS) showed a rotational orientation of 120° (clockwise, recorded as −). Axis compensation was then calculated to ensure proper alignment of the toric optics with the patient’s refractive astigmatism. For the right eye, the rotational orientation of 105° subtracted from the over-refraction (OR) axis of 100° resulted in an adjusted axis of 005°. For the left eye, the rotational orientation of 120° subtracted from the OR axis of 010° yielded an adjusted axis of 110°. The Optosoft ordering software automatically incorporated these compensations when finalizing the lens order, ensuring accurate optical alignment.

Over-refraction measurements were subsequently refined to achieve optimal visual outcomes. The right eye (OD) exhibited an over-refraction of −1.50 −1.00 × 100, which was adjusted to −1.00 −0.50 × 100, resulting in a best-corrected visual acuity (VA) of 20/20 (6/6). The left eye (OS) demonstrated an over-refraction of +1.00 −0.75 × 010, adjusted to +0.50 −0.50 × 010, also achieving a VA of 20/20 (6/6).

These adjustments confirmed precise toric alignment and stable visual performance in both eyes, supporting the overall success of the scleral lens fitting.

Management and plan

The patient was prescribed the OPTOSOFT IS16 scleral contact lenses for both eyes (OU) in addition to her existing spectacles. She was advised to alternate between contact lens and spectacle wear based on environmental conditions and daily needs.

Patient instructions

The patient was instructed to use PuriLens Plus preservative-free saline solution for lens insertion and to clean and disinfect the lenses daily with MeniCare Pure multipurpose solution. Weekly deep cleaning with Menicon Progent was recommended to remove protein and lipid deposits. The patient was advised to remove the lenses overnight and to avoid lens wear during air travel or in extreme environmental conditions, such as excessive heat, dust, or wind exposure. In addition, the patient was counseled to avoid rubbing the eyes, as mechanical stress could worsen corneal thinning associated with keratoconus.

Follow-up visit #1

Return for contact lens clinic evaluation in three months (RTC 3/12) to reassess fit, ocular health, and vision performance.

Medications

To support ocular surface health and enhance comfort during contact lens wear, the following pharmacologic agents were prescribed. Patanol (Olopatadine HCl 0.1% ophthalmic solution) was initiated at one drop in each eye twice daily for 90 days to manage symptoms of allergic conjunctivitis, including itching, burning, and tearing. In addition, regular use of ocular lubricants was recommended to maintain tear film stability and reduce dryness. The patient was advised to use generic artificial tears one drop in each eye three times daily for 180 days, Systane one drop in each eye three times daily, Optive Fusion one drop in each eye up to five times daily for 360 days, and Systane Ultra Minims one drop in each eye four times daily for 180 days.

Final Contact Lens Specifications

Based on the findings and assessments obtained during the trial fitting, the following scleral lenses were finalized and prescribed.

For the right eye (OD), an OPTOSOFT IS16 lens was selected with keratometry value K10, sagittal depth of 5100 µm, base curve of 7.48 mm, and overall diameter of 16.00 mm. The final lens power was −0.75 − 0.50 × 100, providing a best-corrected visual acuity of 20/20. Lens rotation was observed at 105°, with stable alignment and no signs of decentration.

For the left eye (OS), an OPTOSOFT IS16 lens was prescribed with keratometry value K5, sagittal depth of 4400 µm, base curve of 8.05 mm, and overall diameter of 16.00 mm. The final lens power was +0.50 − 0.50 × 010, achieving a best-corrected visual acuity of 20/20. Lens rotation was noted at 120°, demonstrating stable positioning and appropriate scleral alignment.

The finalized parameters yielded excellent visual performance and lens stability bilaterally, confirming optimal fit and visual rehabilitation.

Comments

The scleral lenses (OU) demonstrated a clinically optimal fit. Both apical and limbal clearance were within the desired ranges, ensuring complete corneal protection and limbal safety. The scleral landing zone showed uniform alignment, providing stability with minimal movement. Fluorescein assessment confirmed even tear distribution across the corneal surface. Both lenses remained well-centered with negligible lag or decentration during blink cycles. The patient reported excellent comfort and satisfaction. Best-corrected visual acuity improved to 20/20 in the right eye (OD) and 20/20 in the left eye (OS), confirming successful optical and physiological performance.

## Discussion

In evaluating a 24-year-old patient with suspected keratoconus, several differential diagnoses must be considered, particularly conditions presenting with irregular astigmatism and corneal thinning. The most likely diagnosis is keratoconus, a progressive, non-inflammatory corneal ectasia that typically manifests in adolescence or early adulthood. It is characterized by paracentral thinning with cone-shaped corneal protrusion, resulting in irregular astigmatism and visual distortion. Clinical signs may include a Fleischer ring, Vogt striae, and a scissoring reflex on retinoscopy. Confirmation is obtained with corneal tomography demonstrating inferior steepening and posterior elevation [7].

A milder variant, forme fruste keratoconus, should also be considered, especially when there are no overt clinical signs but topographic or tomographic abnormalities are present. This is often detected during refractive surgery screening and requires elevation-based imaging for accurate diagnosis [8]. Another important differential is pellucid marginal degeneration (PMD), which presents with inferior peripheral corneal thinning and high against-the-rule astigmatism. The classic “crab claw” pattern on corneal topography distinguishes it from keratoconus, as PMD lacks a central cone and the thinning is more peripheral [9].

In patients who wear rigid gas-permeable or soft contact lenses, contact lens-induced corneal warpagemay mimic keratoconus. This is a reversible distortion of corneal shape that resolves upon discontinuation of lens wear and can be confirmed by serial topography [10]. Post-refractive surgery ectasia, such as following LASIK or PRK, can also resemble keratoconus but is typically associated with a history of recent surgery and ectatic changes centered on the ablation zone [11].

Although less common in this age group, keratoglobus should be considered, particularly in cases of diffuse, symmetric thinning with a globular corneal contour. This rare congenital ectasia is often associated with systemic connective tissue disorders [12]. Similarly, Terrien’s marginal degeneration, a non-inflammatory peripheral thinning disorder more common in older males, may present with irregular astigmatism and superficial vascularization, although it is unlikely in a young female [13].

Non-ectatic causes such as ocular surface disease (e.g., severe dry eye) can lead to irregular astigmatism and topographic changes, but do not involve stromal thinning. Diagnosis is confirmed with tear film evaluation and clinical examination [14]. Finally, early corneal scarring from previous trauma, infection (e.g., herpes simplex), or inflammation may present with localized thinning and topographic irregularity. A detailed history and slit-lamp examination can help differentiate these findings from true ectasia [15].

This case highlights the value of custom scleral lenses in the management of advanced keratoconus, particularly in post-CXL eyes [16]. Accurate estimation of sagittal height based on Kmax and corneal tomography enables more predictable lens selection and fitting [17]. In this case, the Scleral lenses restored visual acuity to 6/6 (20/20) in both eyes, confirming the effectiveness of a structured fitting protocol and individualized lens parameter customization [18].

Fitting success was supported by optimal apical and limbal clearance, appropriate scleral alignment, and rotational compensation. These fitting characteristics are critical for minimizing complications such as hypoxia, midday fogging, and conjunctival prolapse [19]. Additionally, the patient’s prior intolerance to RGP lenses and history of CXL are consistent with known indicators for scleral lens suitability [19].

Scleral lenses also provide important long-term therapeutic benefits by protecting the cornea from mechanical trauma, maintaining a stable tear reservoir over the ocular surface, and improving vision-related quality of life. Although surgical interventions remain necessary in end-stage disease, scleral lenses such as the OPTOSOFT IS16 offer a non-invasive, reversible, and effective option for visual rehabilitation in severe keratoconus [20].

During slit-lamp examination, minor blanching was noted; the fact that adjustments were made and the final prescription was dispensed indicates that the fit was optimized for this patient. The literature supports that minor, non-persistent blanching does not necessarily contraindicate a scleral lens fit. Significant blanching under the landing zone indicates a poor fit, but in this case, the minor blanching is acceptable because the +1 adjustment was made.

Despite the successful outcome in this case, ongoing follow-up remains essential to ensure the long-term safety and stability of scleral lens wear. Without regular review, an initially safe clearance could decrease to the point of corneal touch or insufficient limbal protection. Follow-up appointments also provide an opportunity to evaluate conjunctival responses: while only minor blanching was observed and corrected with an adjustment in this case, localized compression or vascular compromise can develop over time and may warrant further lens modification [19].

Regular follow-up visits are critical for monitoring corneal physiology. Studies have shown that larger tear reservoirs (>600 µm) can induce mild, reversible corneal edema, even when thickness remains within normal limits [19]. Regular pachymetry or slit-lamp evaluation ensures that hypoxic stress remains transient and does not progress to chronic complications such as neovascularization. Equally important, follow-up supports the patient’s adaptation to lens wear by addressing common issues such as midday fogging, handling difficulties, or reduced comfort with prolonged wear [19,20].

In summary, scheduled follow-up visits are not optional but are a critical safeguard in scleral lens prescribing. They confirm that the initial fitting remains stable after settling, allow early identification of mechanical or hypoxic complications, and reinforce patient education. By integrating structured follow-up into the management plan, clinicians ensure that the benefits of scleral lenses corneal protection, enhanced optical performance, and improved quality of life are maintained safely over the long term. 

## Conclusions

Scleral lenses, such as the OPTOSOFT IS16, are essential tools for restoring visual function in keratoconus, particularly in highly irregular corneas. This case demonstrates that, with precise diagnostics and a systematic fitting approach, excellent visual and physiological outcomes can be achieved even in advanced cases. It also highlights the importance of individualized lens selection, careful assessment of lens fit, patient education, and regular follow-up to ensure long-term comfort, corneal health, and stable visual performance. Overall, scleral lenses remain a reliable and effective option for the visual rehabilitation of patients with advanced keratoconus.

## References

[1] Mohamed ZD (2025). Keratoconus in Eastern Mediterranean Region: prevalence and risk factors. Afr Vis Eye Health.

[2] Alqudah N, Alqudah A, Alshamarti S, Shannak Z (2025). Evaluating the efficacy and safety of iontophoresis-assisted corneal cross-linking compared to standard CXL in the treatment of keratoconus. Indian J Ophthalmol.

[3] Noushad B, Mohamed Z, Suresh Vankudre G, Hussaindeen JR, Rani K, Elhaj M, Alshamli N (2024). Profile of contact lens prescribing in GCC countries. Cont Lens Anterior Eye.

[4] Hassan A, Abdel-Radi M, Mohammed HM (2025). Causes of visual impairment among patients applying for the visual disability certificate in Upper Egypt: a retrospective study. J Egypt Ophthalmol Soc.

[5] Mushtaq A, Alvi I (2025). Long-term effectiveness of scleral lens treatment in the management of keratoconus: a systematic review. Cureus.

[6] Deshmukh R (2024). False positive Belin-Ambrosio enhanced ectasia screening in small white-to-white diameter. Eye (Lond).

[7] Rabinowitz Y (1998). Keratoconus. Surv Ophthalmol.

[8] Belin MW, Khachikian SS (2009). An introduction to understanding elevation-based topography: how elevation data are displayed - a review. Clin Exp Ophthalmol.

[9] Sridhar MS (2018). Anatomy of cornea and ocular surface. Indian J Ophthalmol.

[10] Michaud L, van der Worp E, Brazeau D, Warde R, Giasson CJ (2012). Predicting estimates of oxygen transmissibility for scleral lenses. Cont Lens Anterior Eye.

[11] Randleman JB, Woodward M, Lynn MJ, Stulting RD (2008). Risk assessment for ectasia after corneal refractive surgery. Ophthalmology.

[12] Wallang BS, Das S (2013). Keratoglobus. Eye (Lond).

[13] Ding Y, Murri MS, Birdsong OC, Ronquillo Y, Moshirfar M (2019). Terrien marginal degeneration. Surv Ophthalmol.

[14] Maeda N, Klyce SD, Smolek MK (1995). Comparison of methods for detecting keratoconus using videokeratography. Arch Ophthalmol.

[15] Lobo AM, Agelidis AM, Shukla D (2019). Pathogenesis of herpes simplex keratitis: the host cell response and ocular surface sequelae to infection and inflammation. Ocul Surf.

[16] Visser ES, Soeters N, Tahzib NG (2015). Scleral lens tolerance after corneal cross-linking for keratoconus. Optom Vis Sci.

[17] Harthan J, Shorter E, Nau C, Nau A, Schornack MM, Zhuang X, Fogt J (2019). Scleral lens fitting and assessment strategies. Cont Lens Anterior Eye.

[18] Ozek D, Kemer OE, Altiaylik P (2018). Visual performance of scleral lenses and their impact on quality of life in patients with irregular corneas. Arq Bras Oftalmol.

[19] Walker MK, Bergmanson JP, Miller WL, Marsack JD, Johnson LA (2016). Complications and fitting challenges associated with scleral contact lenses: a review. Cont Lens Anterior Eye.

[20] Otchere H, Jones L, Sorbara L (2018). The impact of scleral contact lens vault on visual acuity and comfort. Eye Contact Lens.

